# Habitat-driven variation in mycorrhizal communities in the terrestrial orchid genus *Dactylorhiza*

**DOI:** 10.1038/srep37182

**Published:** 2016-11-24

**Authors:** Hans Jacquemyn, Michael Waud, Vincent S. F. T. Merckx, Rein Brys, Daniel Tyteca, Mikael Hedrén, Bart Lievens

**Affiliations:** 1 KU Leuven, Department of Biology, Plant Conservation and Population Biology, B-3001 Leuven, Belgium; 2 KU Leuven, Campus De Nayer, Department of Microbial and Molecular Systems (M^2^S), Laboratory for Process Microbial Ecology and Bioinspirational Management (PME&BIM), B-2860 Sint-Katelijne-Waver, Belgium; 3 Naturalis Biodiversity Center, Leiden University, Leiden, the Netherlands; 4 Biodiversity Research Centre (BDIV), Université Catholique de Louvain, 1348 Louvain-la-Neuve, Belgium; 5 Department of Biology, Biodiversity, Lund University, Sölvegatan 37, S-22362 Lund, Sweden

## Abstract

Orchid species are critically dependent on mycorrhizal fungi for completion of their life cycle, particularly during the early stages of their development when nutritional resources are scarce. As such, orchid mycorrhizal fungi play an important role in the population dynamics, abundance, and spatial distribution of orchid species. However, less is known about the ecology and distribution of orchid mycorrhizal fungi. In this study, we used 454 amplicon pyrosequencing to investigate ecological and geographic variation in mycorrhizal associations in fourteen species of the orchid genus *Dactylorhiza*. More specifically, we tested the hypothesis that variation in orchid mycorrhizal communities resulted primarily from differences in habitat conditions where the species were growing. The results showed that all investigated *Dactylorhiza* species associated with a large number of fungal OTUs, the majority belonging to the Tulasnellaceae, Ceratobasidiaceae and Sebacinales. Mycorrhizal specificity was low, but significant variation in mycorrhizal community composition was observed between species inhabiting different ecological habitats. Although several fungi had a broad geographic distribution, Species Indicator Analysis revealed some fungi that were characteristic for specific habitats. Overall, these results indicate that orchid mycorrhizal fungi may have a broad geographic distribution, but that their occurrence is bounded by specific habitat conditions.

Since the early discoveries that orchid seeds require a fungus to germinate^1^,^2^, it has become more and more clear that orchid mycorrhizal fungi are determining factors that not only drive the local abundance and dynamics of individual orchid populations, but also impact on coexistence and the regional distribution of orchid species^3^. Changes in the composition, richness or abundance of orchid mycorrhizal fungi can therefore be expected to have profound effects on orchid fitness and as a result on orchid distribution^4^,^5^ and community composition^6^. However, little is known about the specific factors that affect the distribution and occurrence of orchid mycorrhizal fungi in natural environments^3^. Recent studies on various types of mycorrhizal fungi have shown that apart from chance events and dispersal limitation^7^,^8^, variation in mycorrhizal communities can also result from differences in local environmental conditions^7^,^9^, often leading to correlated changes in mycorrhizal and plant communities across environmental gradients or distinct habitats^10^.

At present, there are relatively few empirical data on the factors driving variation in orchid mycorrhizal community composition across strong environmental gradients. Recent studies have shown that orchid mycorrhizal communities can vary substantially between species within sites^6^,^11^. In a similar way, mycorrhizal communities have been shown to vary between sites within species^12^,^13^. In this case, variation in mycorrhizal communities was significantly driven by variation in environmental conditions, which suggests that local growth conditions can impact on fungal communities. Further evidence for local environmental conditions affecting orchid mycorrhizal communities comes from comparisons of mycorrhizal communities across different habitats within a single orchid species. For example, it was recently shown that individuals from grassland populations of *Neottia ovata* associated with significantly different fungal communities than individuals from forest populations^14^. Similarly, pronounced differences in mycorrhizal communities were reported between dune slack and forest populations of *Epipactis* species. Overall, these results indicate that differences in habitat conditions can affect the occurrence of particular orchid mycorrhizal fungi and therefore impact on plant-fungus interactions in orchids^15^.

In this study, we investigated differences in mycorrhizal communities among 14 species of the orchid genus *Dactylorhiza*. The genus *Dactylorhiza* consists of a large group of species that are widely distributed across the boreal and temperate zones of Europe, Asia, North America and Northern parts of Africa. Species of the genus *Dactylorhiza* occupy a wide range of habitats with contrasting soil conditions, including acid peat bogs, wet alkaline grasslands, dry meadows and forests^16^,^17^,^18^. Previous research has shown that mycorrhizal specificity in the genus is low and that most *Dactylorhiza* species commonly associate with a wide range of fungi of the Tulasnellaceae^19^,^20^,^21^,^22^. However, members of the Ceratobasidiaceae may also be sporadically observed^19^,^23^. Compared to other European orchid genera, *Dactylorhiza* is unusual in that it contains a large number of species with varying ploidy levels, including diploids, triploids, autotetraploids and a vast number of allotetraploid species^18^,^24^,^25^. The latter comprise groups of species whose origin stemmed from independent hybridization events occurring in various parts of Europe and the Mediterranean Basin. Because previous research has shown that orchid mycorrhizal communities may be significantly affected by ploidy level^26^, differences in ploidy level should be taken into account when investigating variation in mycorrhizal communities in orchids. Moreover, given that allopolyploid species carry the genomes of two different diploid progenitors, it is not unreasonable to assume that differences in ploidy level not only affect mycorrhizal associations, but that genome composition impacts on the mycorrhizal communities associating with orchid species as well.

To determine the relative importance of ploidy level and habitat conditions in determining mycorrhizal communities, we sampled fourteen *Dactylorhiza* species that were characterized by different ploidy levels and occupied distinct habitats, including Mediterranean grasslands and forests (*D. sambucina, D. romana, D. insularis* and *D. markusii*), peat bogs (*D. sphagnicola, D. maculata*), wetlands (*D. fuchsii, D. majalis, D. viridis, D. elata*), coastal habitats (*D. praetermissa, D. incarnata*) and alpine-boreal habitats (*D. alpestris* and *D. lapponica*). More specifically, we asked whether:

1. orchid species from different habitats associated with different mycorrhizal communities;
2. there were specific fungal OTUs that were significantly associated with a particular habitat type;
3. differences in ploidy level or genome composition had a significant impact on mycorrhizal communities.

## Results

### Fungal identity

**Figure 1:**
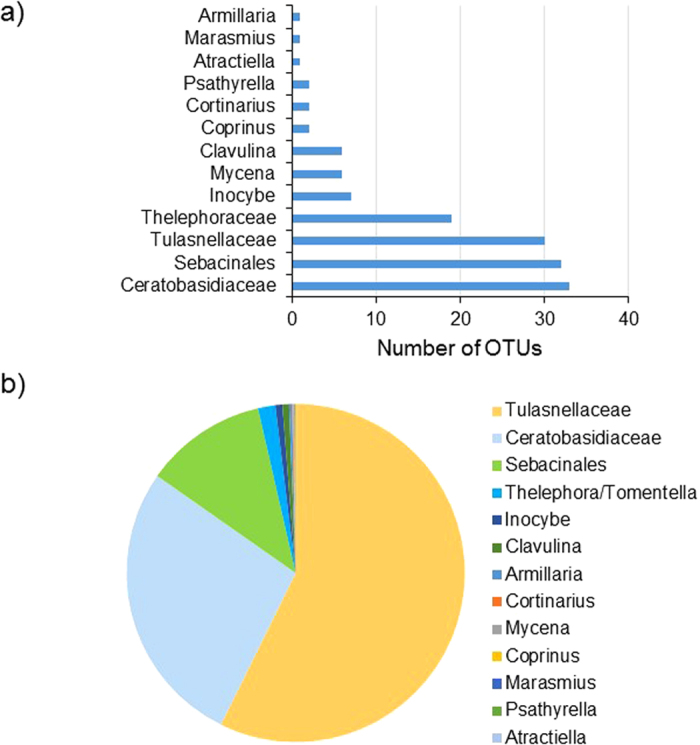
Diversity of putative orchid mycorrhizal fungi detected in the roots of fourteen *Dactylorhiza* species sampled in 35 populations across Europe. (**a**) The number of OTUs belonging to different orchid families/genera. (**b**) Pie chart displaying the frequency distribution of sequences belonging to the different families/genera.

**Figure 2:**
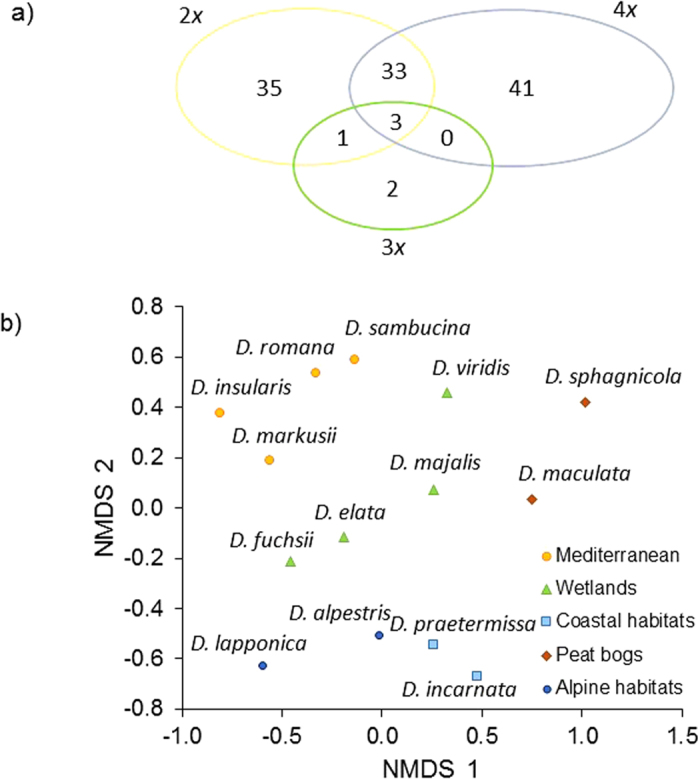
Partitioning of mycorrhizal communities detected in fourteen different *Dactylorhiza* species sampled in 35 populations across Europe. (**a**) Venn diagram showing the number of OTUs that are shared between diploid (2*x*), triploid (3*x*) and tetraploid (4*x*) *Dactylorhiza* species. (**b**) Nonmetric multidimensional scaling (NMDS) plot of mycorrhizal fungi. Each point denotes a different *Dactylorhiza* species. Different colors denote the habitats from which the species were sampled.

After exclusion of global singletons and doubletons, a total of 522 OTUs (94511 sequences) were retrieved, of which 115 (65754 sequences – 68.3% of all sequences) were considered as putative species of orchid mycorrhizal fungi (Table S1). Most orchid mycorrhizal fungi were related to members of the Tulasnellaceae (30 OTUs – 38402 sequences), Ceratobasidiaceae (33 OTUs – 18408 sequences) and Sebacinales (32 OTUs – 7835 sequences) (Fig. 1a,b). Besides a large number of ectomycorrhizal fungi related to *Thelephora/Tomentella* (19 OTUs) was also found, but the number of sequences (1106 sequences – 1.16% of the total number of sequences) was much lower (Fig. 2a,b). Additionally, some members of the fungal genera *Armillaria* (1 OTU), *Atractiella* (1 OTU), *Clavulina* (6 OTUs), *Coprinus* (2 OTUs), *Cortinarius* (2 OTUs) *Inocybe* (7 OTUs), *Marasmius* (1 OTU), *Mycena* (6 OTUs), and *Psathyrella* (2 OTUs) were sporadically observed (Fig. 1a), but in all cases the number of sequences was low (Fig. 1b). Because the mycorrhizal status of these fungi is doubtful, they were not considered in all subsequent analyses.

Phylogenetic analyses of the Tulasnellaceae OTUs showed that they belonged to three well-supported clades (Fig. S1). Clades A and B have been previously shown to be mycorrhizal in orchids^27^, but the distantly related clade C appears to consist of a range of *Tulasnella* sequences that have only rarely been retrieved from orchids. All Ceratobasidiaceae OTUs belonged to a well-supported clade of closely related OTUs (Fig. S2), in which no strong subdivision was present. As to the Sebacinales, two major clades (Clade A and B) could be discerned corresponding to the two major groups with different ecological characteristics identified before^28^,^29^ (Fig. S3). In total, 9 OTUs (4350 sequences) were assigned to clade A and 23 OTUs (2592 sequences) were assigned to clade B (Fig. S3). However, only one OTU (OTU1) of clade A had a high sequence number and this OTU was exclusively retrieved in the alpine-boreal species *D. lapponica* and *D. alpestris*. Within the Thelephoraceae (Fig. S4), OTUs were found to be part of a well-supported clade, which included several *Tomentella* and *Thelephora* representatives. No further subdivision could be made.

### Fungal diversity and association patterns

All *Dactylorhiza* species associated with a large number of orchid mycorrhizal fungi, most often members of the Tulasnellaceae and Ceratobasidiaceae, although members of the Thelephoraceae and Sebacinales were also frequently recovered. The total number of OTUs per orchid species varied between 6 (*D. insularis*) and 34 (*D. fuchsii*) (average: 17 OTUs per species). However, the majority of fungi was found in only one or two orchid species, indicating large turnover in mycorrhizal communities between the studied orchid species. Only one OTU (OTU4), which belonged to the Tulasnellaceae, occurred in more than half of the sampled orchid species. Two OTUs were found in half of the sampled species, one of which (OTU3) belonged to the Tulasnellaceae, while the other (OTU60) belonged to the Ceratobasidaceae. Four OTUs were detected in six *Dactylorhiza* species, three (OTU9, OTU10 and OTU18) of which belonged to the Tulasnellaceae and one (OTU11) to the Ceratobasidiaceae.

Within individual populations, a large number of mycorrhizal fungi was detected, confirming previous results that species of the genus *Dactylorhiza* show low specificity towards mycorrhizal fungi^22^. On average, nine different (range: 2–22) mycorrhizal OTUs were found in a population. Members of the Tulasnellaceae were detected in almost every population, but the number of OTUs was low in *D. viridis*, which mainly associated with members of the Ceratobasidiaceae. Members of the Ceratobasidiaceae were absent in populations of *D. insularis*. Sebacinales fungi were absent in populations of the Mediterranean species *D. markusii* and *D. insularis*, which primarily associated with *Tulasnella* and *Thelephora* OTUs. Finally, no fungi of the Thelephoraceae were observed in populations of *D. maculata* and *D. viridis* (Fig. S5).

Of the 115 detected OTUs that were considered orchid mycorrhizal, 77 OTUs were found in tetraploid species, 72 OTUs in diploid species and 6 OTUs in the triploid species (Fig. 2a). Three of these orchid mycorrhizal OTUs were shared between diploid, triploid and tetraploid species. Diploid and tetraploid species shared a total of 38 OTUs (Fig. 2a). The NMDS plot showed no clear differences between diploid and polyploid *Dactylorhiza* species, which was confirmed by the permanova analysis (pseudo-*F* = 1.158; *P* = 0.28) (Fig. S6). However, when plotting the habitat from which orchid species were sampled on the NMDS plot, clear differences between species from different habitats became apparent (Fig. 2b). These results were confirmed by the permanova analysis, which showed significant differences (pseudo-*F* = 2.58; *P* = 0.001) in mycorrhizal communities between species from different habitats. Species from Mediterranean habitats were clearly separated from the other groups at the upper part of the plot. Species sampled from peat bogs (*D. maculata* and D. *sphagnicola*) were located at the right-hand part of the plot, whereas species from wetlands were located somewhere intermediate between these two. Species from alpine-boreal habitats clustered together at the left hand site of the plot, whereas species from coastal habitats clustered together at the lower right hand side of the plot.

**Figure 3:**
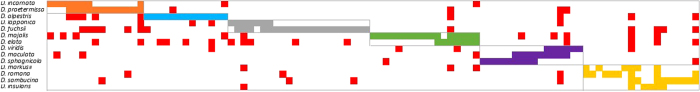
Matrix representation of the studied orchid mycorrhizal network encompassing fourteen *Dactylorhiza* species (rows) and putative orchid mycorrhizal operational taxonomic units (OTUs) (columns). Different colors represent different modules. Red cells are species links gluing the six modules together into a coherent network, and non-red cells are links within modules.

The modularity analysis showed that the network of interactions was significantly modular (*M*_obs_ = 0.4737, *M*_random_ = 0.4461 ± 0.0084, *P* < 0.001) and that six modules were identified (Fig. 3). The largest module consisted of four species and contained the four sampled species typically occurring in Mediterranean habitats (*D. markusii, D. insularis, D. romana* and *D. sambucina*) (Fig. 3). *D. praetermissa* and *D. incarnata* formed another module and represent species that were mainly sampled in coastal habitats. The module of *D. maculata, D. sphagnicola* and *D. viridis* showed less links to other modules. Finally, *D. alpestris* formed a module that consisted of a single species (Fig. 3). Broadly speaking, these modules can also be brought back to the habitats from which the orchids were sampled, but not to their ploidy level.

Finally, Species Indicator Analysis identified three OTUs that were significantly associated with peat bogs (OTU5, OTU25 and OTU420), one OTU that significantly associated with alpine boreal habitats (OTU1) and two that associated with wetlands and Mediterranean habitats (OTU4 and OTU60). No OTUs were identified that significantly associated with a particular ploidy level.

## Discussion

In this study, we investigated the mycorrhizal communities associating with a large number of species of the orchid genus *Dactylorhiza*. Consistent with previous research, our results showed that species of the genus *Dactylorhiza* associated with a large suite of mycorrhizal fungi^21^,^22^. These results also confirm recent and more general observations that showed a high diversity of fungi associating with orchids in comparison with other mycorrhizal systems (arbuscular mycorrhizal fungi, ectomycorrhizal fungi)^30^. The most abundant fungi were typical rhizoctonia fungi from the families Tulasnellaceae, Ceratobasidiaceae and Sebacinales. Members of the Tulasnellaceae and Ceratobasidiaceae have been repeatedly shown to associate with terrestrial temperate orchids from Europe^27^,^31^,^32^. Our results further confirm previous analyses that have shown that members of the Tulasnellaceae are the prime symbionts in *Dactylorhiza*^20^,^21^,^22^,^23^. The number of *Tulasnella* OTUs per species that was detected in an earlier study on *Tulasnella* diversity in five *Dactylorhiza* species^22^ was comparable with the numbers reported here for the same set of species (average: 6.4 ± 1.5 and 7.8 ± 3.4, respectively), despite fewer populations and individuals studied per species in the current study. At least seven *Tulasnella* OTUs (70%) showed strong genetic resemblance to previously detected OTUs, indicating that, we have picked up the majority of strains that were found associating with *Dactylorhiza* before. Besides, a considerable percentage of sequences belonged to OTUs of Sebacinales, most often members of clade B, although some representatives of clade A were also identified. Sebacinales of clade A are mainly ectomycorrhizal fungi that associate with trees and mycoheterotrophic orchids, whereas Sebacinales of clade B have been shown to be mycorrhizal fungi in Ericaceae, green orchids and liverworts^28^,^29^. However, recent research on the green orchid *Neottia ovata* has shown that Sebacinales of group A can be observed associating with green orchids as well^13^,^33^. Interestingly, Sebacinales of clade A were only retrieved from *D. alpestris, D. lapponica* and *D. fuchsii*, but not in the other species, suggesting that these fungi may be limited to particular habitats.

Besides these rhizoctonia fungi, we found a large number of ectomycorrhizal fungi of the Thelephoraceae. These fungi have previously been shown to associate with orchids that typically occur in forests. For example, the major fungi associating with the mycoheterotrophic *Corallorhiza odonthorhiza* were *Tomentella* fungi^34^. In the forest orchids *Cephalanthera damasonium* and *C. longifolia* several members of the Thelephoraceae, including *Tomentella* and *Pseudotomentella*, were found in germinating seeds^35^ (Bidartondo & Read 2008). Members of the Thelephoraceae were also detected in the forest orchid *Neottia ovata*^13^,^33^. However, recent investigations of a large number of terrestrial orchids in Mediterranean grasslands showed that associations with ectomycorrhizal fungi are not restricted to woodland orchids and that grassland orchids may also commonly associate with members of the Thelephoraceae^36^. Our results are clearly in line with these observations and suggest that these fungi may play a role in the life cycle of grassland orchids as well.

Although the precise factors driving local variation in fungal community structure and plant-fungal interactions remain largely unclear, there is mounting evidence that variation in local environmental conditions can generate pronounced differences in mycorrhizal communities^9^,^10^. Our results seem to confirm this as species from similar habitats clearly clustered together in the NMDS plot, whereas the occurrence of significant modularity indicates little overlap in mycorrhizal communities between species from different habitats. Recent research in the orchid genus *Epipactis* has shown similar habitat-dependent variation in mycorrhizal communities^15^. The co-occurring, but distantly related species *E. neerlandica* and *E. palustris* had much more fungi in common with each other than with *E. helleborine* that occurred in forests and from which *E. neerlandica* has been derived^15^. On the other hand, these findings are in contrast with a similar large-scale study on *Orchis*^31^,^32^, where no modularity was observed. However, in *Orchis* there was much less variation in habitats since most *Orchis* species typically occur in dry, nutrient-poor grasslands or forest edges with high pH.

The possibility that our results were partly the result of geographic proximities cannot be fully ruled out. However, the four species from the *D. sambucina* group that were studied here, were sampled across distances that were much larger than the ones separating wetland habitats, suggesting geographic proximity probably had a smaller impact on mycorrhizal communities. The closely related *D. alpestris* and *D. lapponica* clustered together in the NMDS plot and typically occur in boreal-alpine environments, but were sampled at sites that were more than 1500 km apart (alkaline seepages in the French Alps (*D. alpestris*) and boggy woods in Sweden (*D. lapponica*)). Individuals of *D. sphagnicola* and *D. maculata* were mainly sampled in strongly acidic peat bogs. Our results further showed that some of the retrieved fungi may be habitat generalists and have large geographic distribution ranges, since they were isolated from *Dactylorhiza* species from different habitat types and at fairly large distances from each other, whereas others may be habitat specialists. Indicator Species Analysis showed that at least three different fungi were significantly associated with peat bogs. Two Tulasnella fungi were also characteristic for Mediterranean habitats.

Apart from differences in habitat conditions, variation in mycorrhizal communities can also be shaped by differences in ploidy level^26^. Polyploids, for example, have been shown to be less dependent on mycorrhizal fungi, which play a substantial role in plant nutrient acquisition and coping with abiotic stress, than diploids, because they generally show a higher tolerance to nutrient stress, drought, cold, or salinity^37^. However, we found no significant differences in mycorrhizal community composition between diploid, triploid and tetraploid species and no fungal OTUs were identified that were characteristic for a particular ploidy level. Diploid and tetraploid species shared a large number of mycorrhizal fungi and the NMDS showed that there was no overall significant difference in fungal community composition between diploid, triploid and tetraploid species. These results contrast with findings of Těšitelová *et al*.^26^, who showed that diploid and tetraploid individuals sampled from different populations shared very few OTUs. Our results further indicated that variation in mycorrhizal communities was also not clearly related to the genomic composition of the orchids (see also Fig. S6). Although species of the *D. sambucina* group clearly separated from the other groups at the upper part of the plot, for the other species the distinction was less clear. Allotetraploids of *D. fuchsii* by *D. incarnata* origins were located somewhere intermediate between the two parental genomes, but the dispersion of allotetraploids along the axis perpendicular to the separation of *D. fuchsii* and *D. incarnata* was fairly large. Mycorrhizal communities of allotetraploids with *D. maculata* rather than *D. fuchsii* origins (*D. sphagnicola* and *D. elata*) were also not clearly related to each other, mainly due to the unclear position of *D. elata*, although *D. maculata* and *D. sphagnicola* tended to depart in the same direction.

Overall, our results showed that all sampled *Dactylorhiza* species associated with a large number of mycorrhizal OTUs encompassing multiple fungal genera, confirming previous research that mycorrhizal specificity in European terrestrial orchids tends to be low. Moreover, they also showed that ploidy level had no major impact on mycorrhizal communities in the sampled *Dactylorhiza* species. However, there was a clear and significant relationship between the habitats from which the orchid species were sampled and mycorrhizal community composition, suggesting that variation in mycorrhizal associations in orchid species is to some extent controlled by local environmental conditions.

## Methods

### Study species

The genus *Dactylorhiza* consists of a large group of species that are widely distributed across the boreal and temperate zones of Europe, Asia, North America and Northern parts of Africa and that occupy a wide range of habitats, including peat bogs, wet grasslands, dry meadows and forests^16^,^17^,^18^. Its taxonomical status is quite complex due to high morphological variation of many taxa and the numerous intra- and inter-genus hybrids^18^. Most *Dactylorhiza* species are summergreen with the leafy shoots appearing in early spring and mostly lasting until late summer – beginning of autumn. Observational studies have indicated that germination most likely takes place in late summer – beginning of autumn less than 3 months after seeds have been shed^38^. In agreement with *Gymnadenia* and *Pseudorchis*, but in contrast to the majority of other tuberous terrestrial orchids in Europe, the tubers are lobed or palmately divided. All species investigated so far have been shown to be dependent on mycorrhizal fungi^20^,^21^,^22^,^23^. Mycorrhizal colonization is mainly observed in the slender roots and sometimes in the extremities of the finger-like extensions of the tuber^19^,^38^.

The polyploid members of *Dactylorhiza* studied here have relatively recent origins and their relationships to diploid members of the genus have been elucidated by means of molecular data^17^,^18^,^39^,^40^. The two diploid species *D. fuchsii* and *D. incarnata* have been involved in the formation of most of the polyploid species. To facilitate comparison with polyploid members of the genus, their genomes are annotated with the letters F and I, respectively. The autotetraploid *D. maculata* is related to *D. fuchsii*, but must have originated from a diploid parental species somewhat divergent from the latter^39^,^41^ and its genome may be described as F^M^. The allotetraploid species *D. alpestris, D. lapponica, D. majalis* and *D. praetermissa* all have origins from parents similar to present day *D. fuchsii* and *D. incarnata*, and their genome compostions may thus be given as FFII^17^,^18^. The two allotetraploids *D. sphagnicola* and *D. elata* include one genome similar to that of present-day *D. maculata*^39^,^42^, and are best described by the genome formula F^M^F^M^II, but it should be observed that *D. sphagnicola* is probably considerably younger than *D. elata*^18^,^43^. The three diploids *D. markusii, D. romana*, and *D. sambucina* and the triploid *D. insularis* are more closely related to each other than to any other member of the genus^40^,^44^ and may be described by an S genome. Finally, the diploid *D. viridis* is probably sister to the rest of the genus^45^ and is best described by the genome composition VV.

### Sampling

Sampling took place in May-June of 2010, 2011 and 2012. A total of 38 sites distributed across six European countries (Belgium, France, Italy, Portugal, Sweden and the United Kingdom) were sampled (Fig. S6; Table S2). At each site, root samples were collected, yielding a total of 114 sampled individuals from one to three populations of the 14 selected species. To minimize damage to the populations, three samples per population were taken. The sampled populations occupied a wide range of habitats on both acidic and alkaline soils, including Mediterranean grasslands and forests (*D. sambucina, D. romana, D. insularis* and *D. markusii*), peat bogs (*D. sphagnicola, D. maculata*), wetlands (*D. fuchsii, D. majalis, D. viridis, D. elata*), coastal habitats (*D. praetermissa, D. incarnata*) and alpine-boreal habitats (*D. alpestris* and *D. lapponica*).

### Molecular assessment of the mycorrhizal fungi

All roots were surface sterilized (30 s submergence in 1% sodium hypochlorite, followed by three 30 s rinse steps in sterile distilled water) and microscopically checked for mycorrhizal colonization. Subsequently, DNA was extracted from 0.5 g mycorrhizal root fragments using the UltraClean Plant DNA Isolation Kit as described by the manufacturer (Mo Bio Laboratories Inc., Solana Beach, CA, USA). Amplicon libraries were created using the primers ITS1OF-C (AACTCGGCCATTTAGAGGAAGT)/ITS1OF-T (AACTTGGTCATTTAGAGGAAGT) and ITS4OF (5′-GTTACTAGGGGAATCCTTGTT-3′)^46^. All individuals per species were assigned unique MID (Multiplex Identifier) barcode sequences according to the guidelines for 454 GS-FLX+ Titanium Lib-L sequencing. Polymerase chain reaction (PCR) amplification was performed in duplicate in a 25 μl reaction volume containing 0.15 mM of each dNTP, 0.5 μM of each primer, 1 U Titanium Taq DNA polymerase, 1X Titanium Taq PCR buffer (Clontech Laboratories, Palo Alto, CA, USA), and 10 μl of DNA extract. PCR conditions were as follows: initial denaturation of 2 min at 94 °C followed by 30 cycles of 45 s at 94 °C, 45 s at 60 °C, and 45 s at 72 °C. After resolving the amplicons by agarose gel electrophoresis, amplicons within the appropriate size range (700 to 1000 bp) were cut from the gel and purified using the Qiaquick gel extraction kit (Qiagen, Hamburg, Germany). Purified dsDNA amplicons were quantified using the Qubit fluorometer (Invitrogen) and pooled in equimolar quantities of 1.00E + 10 molecules per sample, resulting in two amplicon libraries designed with consideration to the recommendations of Lindahl *et al*.^47^, each representing one of the two PCR replicates. The quality of the amplicon libraries was assessed using an Agilent Bioanalyzer 2100 and high sensitivity DNA chip (Agilent Technologies, Waldbronn, Germany). Each amplicon library was loaded onto 1/8^th^ of a 454 Pico Titer Plate (PTP). Pyrosequencing was performed using the Roche GS FLX+ instrument and Titanium chemistry using flow pattern B and software version 2.9 according to the manufacturer’s instructions (Roche Applied Science, Mannheim, Germany).

### Data analysis

#### Fungal diversity and community composition

Sequences obtained from the 454 pyrosequencing run were assigned to the appropriate sample based on both barcode and primer sequences, allowing zero discrepancies, and were subsequently trimmed from the barcodes and primers using CUTADAPT 1.0^48^. As GS-FLX+ instrument flow pattern B minimizes the necessity for post-run denoising, sequences were trimmed based on a minimum Phred score of 30 (base call accuracy of 99.9%) averaged over a 50 bp moving window and sequences with ambiguous base calls or homopolymers longer than 8 nucleotides were rejected, as were chimeric sequences detected by the UCHIME chimera detection program (*de novo* algorithm)^49^. Sequences which passed all quality control procedures were used as the basis for all further analyses. Sequence lengths were set to 350 nucleotides. For further analysis, sequence data obtained for both PCR replicates were combined for each sample.

Operational Taxonomic Units (OTUs) were determined using UPARSE^50^, wherein sequences exceeding 97% sequence homology were clustered into the same OTU. OTUs representing only one or two sequences in the whole dataset (global singletons or doubletons) were removed from further analysis as it has been shown that this improves the accuracy of diversity estimates^51^. The remaining OTUs were assigned taxonomic identities to the highest taxonomic rank possible/family level based on manual screening of the top 10 BLAST^52^ results of representative sequences (as indicated by UPARSE) using GenBank^53^, including uncultured/environmental entries. Although there are reference sequence databases available (e.g. UNITE), preliminary analyses indicated that sequences corresponding to known orchid mycorrhizal sequences were only marginally represented. Finally, OTUs were manually screened for possible orchid-associating mycorrhizal families based on the data provided in Table 12.1 in Dearnaley *et al*.^54^. Only OTUs that were detected on orchid roots and had a high BLAST identity (>90%) to known orchid-associating mycorrhizal families were retained for further analysis.

#### Phylogenetic analyses

Separate phylogenetic analyses were performed with the OTUs that were assigned to Tulasnellaceae, Ceratobasidiaceae and Sebacinales. ITS sequence data of representatives of these groups and appropriate outgroups were downloaded from GenBank. Alignments were build using the MAFFT v.6.814b alignment tool^55^ implemented in Geneious Pro v5.5.6 (Biomatters, New Zealand). The GTR + I + G substitution model was selected to best fit for all four datasets using jModetest 2.1.5^56^ under the Akaike Information Criterion. Phylogenetic analyses were performed under the Maximum Likelihood optimality criterion with RAxML v8.0^57^. Clade support was estimated by non-parametric bootstrap analyses on 500 pseudo-replicate data sets.

#### Ploidy, habitat and mycorrhizal communities

Non-metric multidimensional scaling (NMDS) was used to visualize differences in mycorrhizal communities across species. To test the hypothesis that fungal community composition differed between diploid and polyploid (triploid and tetraploid) species or between habitats, we used multivariate permutational analysis of variance using the adonis function of the vegan package^58^ in R. We also tested the hypothesis that the network of interactions between *Dactylorhiza* species and associating mycorrhizal fungi was significantly modular and that the identified modules can be brought back to differences in ploidy levels or habitats from which the species were sampled. To this end, we used the simulated annealing algorithm developed by Guimerà & Amaral^59^. The algorithm was specifically designed to identify modules whose nodes have the majority of their links inside their own module and provides an index of modularity 
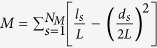
, where *N*_M_ is the number of modules, *L* represents the number of links in the network, *l*_*s*_ is the number of links between nodes in module *s*, and *d*_*s*_ is the sum of the number of links of the nodes in module *s. M* values vary between 0 and −1/*N*_M_ and measure the extent to which species have more links than expected if linkage is random. To determine the significance of the observed modularity index, 999 random networks with the same species degree distribution as the original network were constructed and the observed modularity index was compared with indices from random networks^59^. Finally, we used Species Indicator Analysis to investigate whether some mycorrhizal fungi were significantly associated with a particular habitat type. We used the multipatt function in the R package indicspecies to define indicator species of both individual habitats and combinations of habitats^60^.

## Additional Information

**How to cite this article**: Jacquemyn, H. *et al*. Habitat-driven variation in mycorrhizal communities in the terrestrial orchid genus *Dactylorhiza. Sci. Rep.* **6**, 37182; doi: 10.1038/srep37182 (2016).

**Publisher's note:** Springer Nature remains neutral with regard to jurisdictional claims in published maps and institutional affiliations.

## Supplementary Material

Supplementary Information
