# Supplementary material for: Habitat-driven variation in mycorrhizal communities in the terrestrial orchid genus Dactylorhiza
Source: Sci Rep. 2016 Nov 24;6:37182. doi: 10.1038/srep37182 (PMC5121631; doi:10.1038/srep37182)
Supplement: Supplementary Information [file srep37182-s1.pdf]

Habitat-driven variation in mycorrhizal communities in the terrestrial orchid genus *Dactylorhiza*

Supporting Information

Hans Jacquemyn[1]*, Michael Waud[1,2], Vincent S. F. T. Merckx[3], Rein Brys[1], Daniel Tyteca[4], Mikael Hedrén[5], Bart Lievens[2]

[1]*KU Leuven, Department of Biology, Plant Conservation and Population Biology, B-3001 Leuven, Belgium*

[2]*KU Leuven, Campus De Nayer, Department of Microbial and Molecular Systems (M[2]S), Laboratory for Process Microbial Ecology and Bioinspirational Management (PME&BIM), B-2860 Sint-Katelijne-Waver, Belgium*

[3]*Naturalis Biodiversity Center, Leiden University, Leiden, the Netherlands*

[4]*Biodiversity Research Centre (BDIV), Université Catholique de Louvain, 1348 Louvain-la-Neuve, Belgium*

[5]*Department of Biology, Biodiversity, Lund University, Sölvegatan 37, S-22362 Lund, Sweden*

*Correspondence author. E-mail: hans.jacquemyn@bio.kuleuven.be

Fig. S1 Highest likelihood tree obtained with the Maximum Likelihood analyses of the Tulasnellaceae dataset. Bootstrap support values of ≥50% are shown next to the nodes. Bar represents 0.5 substitutions per site.

Fig. S2 Highest likelihood tree obtained with the Maximum Likelihood analyses of the Ceratobasidiaceae dataset. Bootstrap support values of ≥50% are shown next to the nodes. Bar represents 0.5 substitutions per site.

Fig. S3 Highest likelihood tree obtained with the Maximum Likelihood analyses of the Sebacinales dataset. Bootstrap support values of ≥50% are shown next to the nodes. Bar represents 0.2 substitutions per site.

Fig. S4 Highest likelihood tree obtained with the Maximum Likelihood analyses of the Thelephoraceae dataset. Bootstrap support values of ≥50% are shown next to the nodes. Bar represents 0.3 substitutions per site.

Fig. S5 Number of operational taxonomic units (OTUs) of the four main orchid mycorrhizal families (Tulasnellaceae, Ceratobasidiaceae, Sebacinaceae and Thelephoraceae) obtained from the roots of 117 individuals of fourteen *Dactylorhiza* species collected in 35 populations across Europe.

Fig. S6 Partitioning of mycorrhizal fungi across diploid, triploid and tetraploid *Dactylorhiza* species. Nonmetric multidimensional scaling (NMDS) plot of mycorrhizal fungi detected in fourteen different *Dactylorhiza* species sampled in 35 populations across Europe. Each point denotes a different *Dactylorhiza* species.

Fig. S7 Map showing the locations of all sampled populations of fourteen different *Dactylorhiza* species in Europe. The map was created using ArcGis 9.0 (www.esri.com) and was subsequently edited in Adobe Photoshop (www.adobe.com).

Table S1 List of operational taxonomic units (OTUs) corresponding to orchid-associating mycorrhizal families discovered in this study.

Table S2 Overview of surveyed *Dactylorhiza* species and populations.

Fig. S1

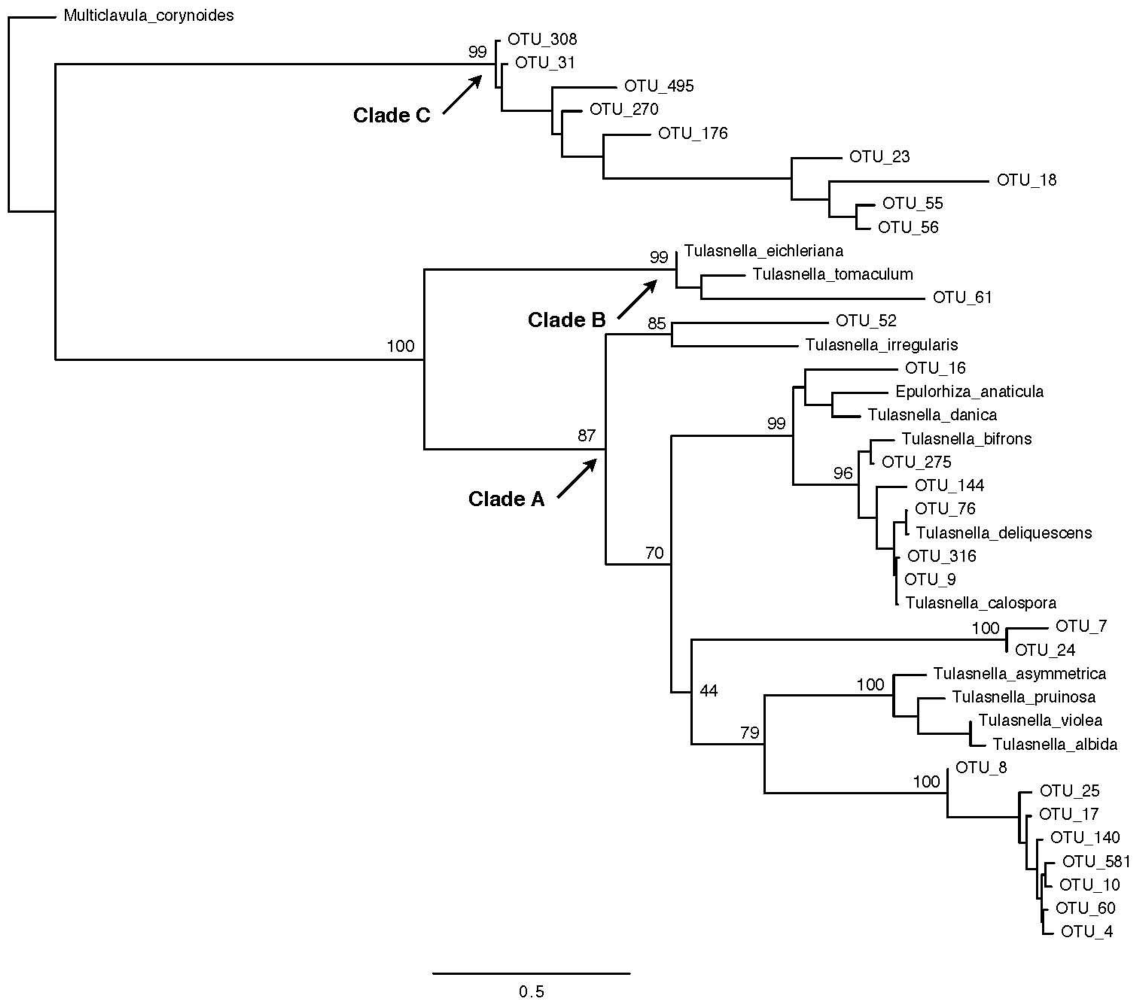
Multiclavula_corynoides
OTU_308
OTU_31
99
OTU_495
OTU_270
OTU_176
Clade C
OTU_23
OTU_18
OTU_55
OTU_56
99
Tulasnella_eichleriana
Tulasnella_tomaculum
OTU_61
Clade B
85
OTU_52
Tulasnella_irregularis
100
OTU_16
Epulorhiza_anaticula
Tulasnella_danica
99
87
Tulasnella_bifrons
OTU_275
96
OTU_144
Clade A
OTU_76
Tulasnella_deliquescens
70
OTU_316
OTU_9
Tulasnella_calospora
100
OTU_7
OTU_24
Tulasnella_asymmetrica
100
Tulasnella_pruinosa
44
Tulasnella_violea
79
Tulasnella_albida
OTU_8
100
OTU_25
OTU_17
OTU_140
OTU_581
OTU_10
OTU_60
OTU_4
0.5

Fig. S2

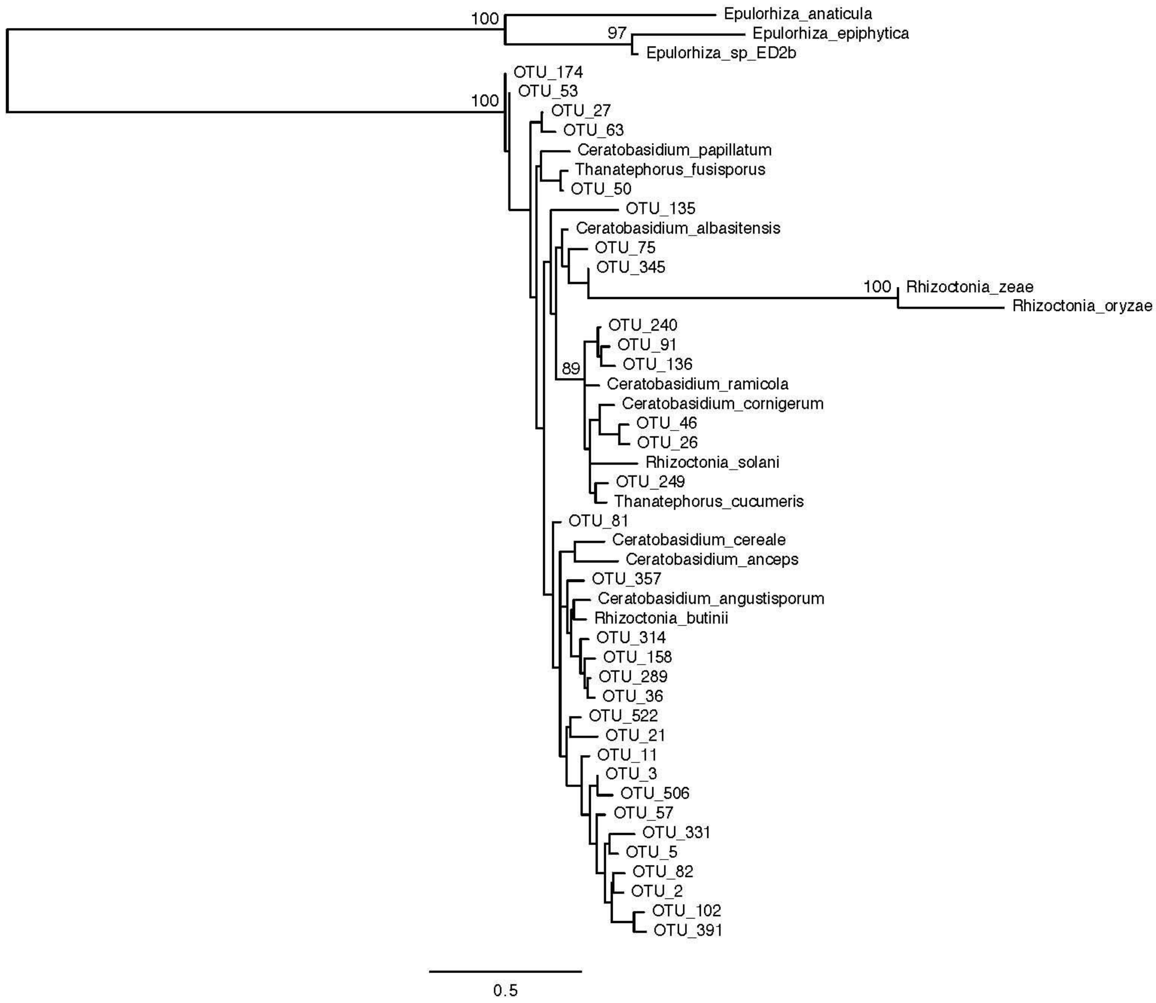
Epulorhiza_anaticula
Epulorhiza_epiphytica
Epulorhiza_sp_ED2b
100
97
OTU_174
OTU_53
100
OTU_27
OTU_63
Ceratobasidium_papillatum
Thanatephorus_fusisporus
OTU_50
OTU_135
Ceratobasidium_albasitensis
OTU_75
OTU_345
100
Rhizoctonia_zeae
Rhizoctonia_oryzae
OTU_240
OTU_91
OTU_136
89
Ceratobasidium_ramicola
Ceratobasidium_cornigerum
OTU_46
OTU_26
Rhizoctonia_solani
OTU_249
Thanatephorus_cucumeris
OTU_81
Ceratobasidium_cereale
Ceratobasidium_anceps
OTU_357
Ceratobasidium_angustisporum
Rhizoctonia_butinii
OTU_314
OTU_158
OTU_289
OTU_36
OTU_522
OTU_21
OTU_11
OTU_3
OTU_506
OTU_57
OTU_331
OTU_5
OTU_82
OTU_2
OTU_102
OTU_391
0.5

Fig. S3

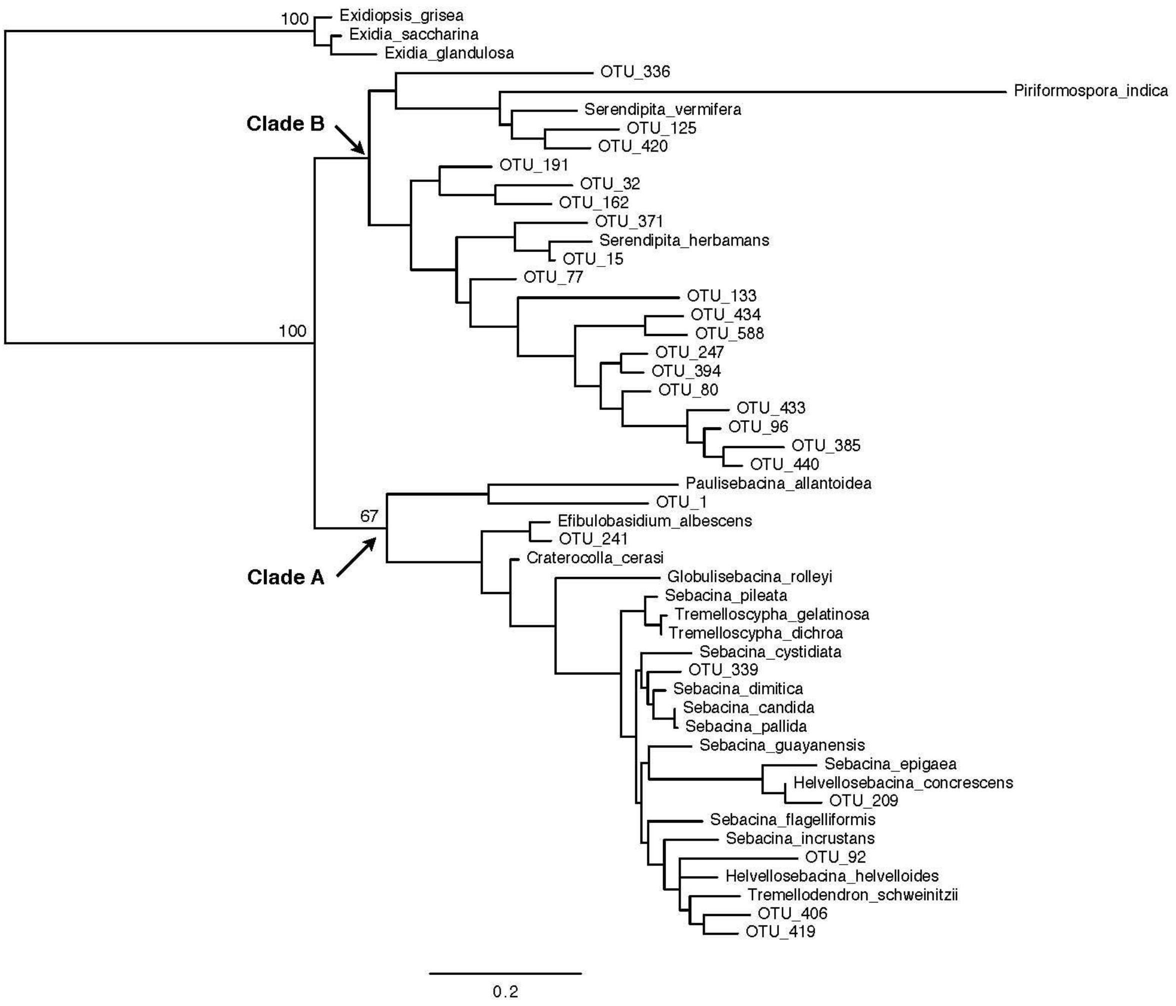
100
Exidiopsis_grisea
Exidia_saccharina
Exidia_glandulosa
OTU_336
Piriformospora_indica
Serendipita_vermifera
OTU_125
OTU_420
Clade B
OTU_191
OTU_32
OTU_162
OTU_371
Serendipita_herbamans
OTU_15
OTU_77
OTU_133
OTU_434
OTU_588
100
OTU_247
OTU_394
OTU_80
OTU_433
OTU_96
OTU_385
OTU_440
Paulisebacina_allantoidea
OTU_1
67
Efibulobasidium_albescens
OTU_241
Craterocolla_cerasi
Clade A
Globulisebacina_rolleyi
Sebacina_pileata
Tremelloscypha_gelatinosa
Tremelloscypha_dichroa
Sebacina_cystidiata
OTU_339
Sebacina_dimitica
Sebacina_candida
Sebacina_pallida
Sebacina_guayanensis
Sebacina_epigaea
Helvellosebacina_concrescens
OTU_209
Sebacina_flagelliformis
Sebacina_incrustans
OTU_92
Helvellosebacina_helvelloides
Tremellodendron_schweinitzii
OTU_406
OTU_419
0.2

Fig. S4

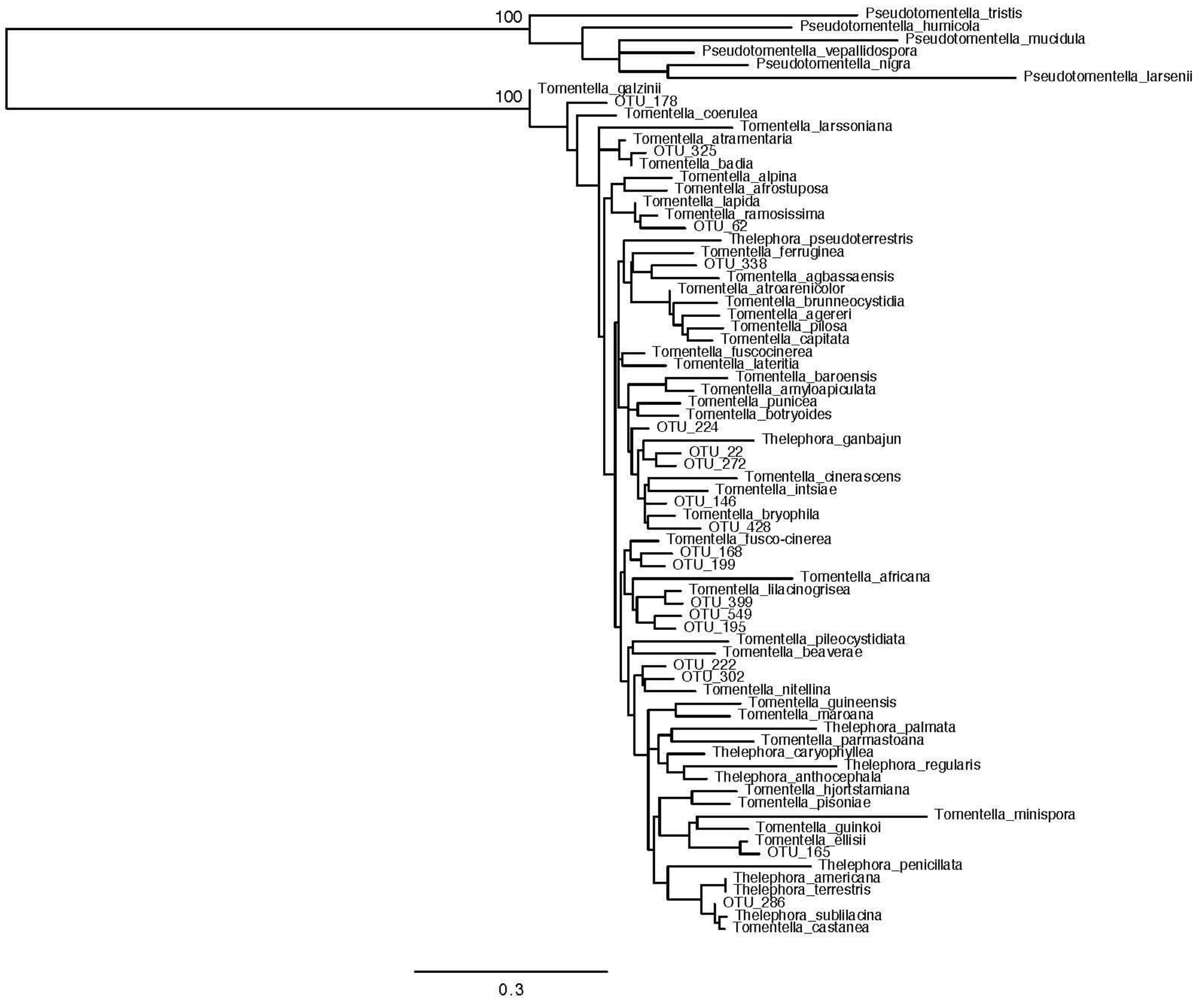
100
Pseudotomentella_tristis
Pseudotomentella_humicola
Pseudotomentella_mucidula
Pseudotomentella_vepallidospora
Pseudotomentella_nigra
Pseudotomentella_larsenii
100
Tomentella_galzinii
OTU_178
Tomentella_coerulea
Tomentella_larssoniana
Tomentella_atramentaria
OTU_325
Tomentella_badia
Tomentella_alpina
Tomentella_afrostuposa
Tomentella_lapida
Tomentella_ramosissima
OTU_62
Thelephora_pseudoterrestris
Tomentella_ferruginea
OTU_338
Tomentella_agbassaensis
Tomentella_atroarenicolor
Tomentella_brunneocystidia
Tomentella_agereri
Tomentella_pilosa
Tomentella_capitata
Tomentella_fuscocinerea
Tomentella_lateritia
Tomentella_baroensis
Tomentella_amyloapiculata
Tomentella_punicea
Tomentella_botryoides
OTU_224
Thelephora_ganbajun
OTU_22
OTU_272
Tomentella_cinerascens
Tomentella_intsiae
OTU_146
Tomentella_bryophila
OTU_428
Tomentella_fusco-cinerea
OTU_168
OTU_199
Tomentella_africana
Tomentella_lilacinogrisea
OTU_399
OTU_549
OTU_195
Tomentella_pileocystidiata
Tomentella_beaverae
OTU_222
OTU_302
Tomentella_nitellina
Tomentella_guineensis
Tomentella_maroana
Thelephora_palmata
Tomentella_parmastoana
Thelephora_caryophyllea
Thelephora_regularis
Thelephora_anthocephala
Tomentella_hjortstamiana
Tomentella_pisoniae
Tomentella_minispora
Tomentella_guinkoi
Tomentella_ellisii
OTU_165
Thelephora_penicillata
Thelephora_americana
Thelephora_terrestris
OTU_286
Thelephora_sublilacina
Tomentella_castanea
0.3

Fig. S5

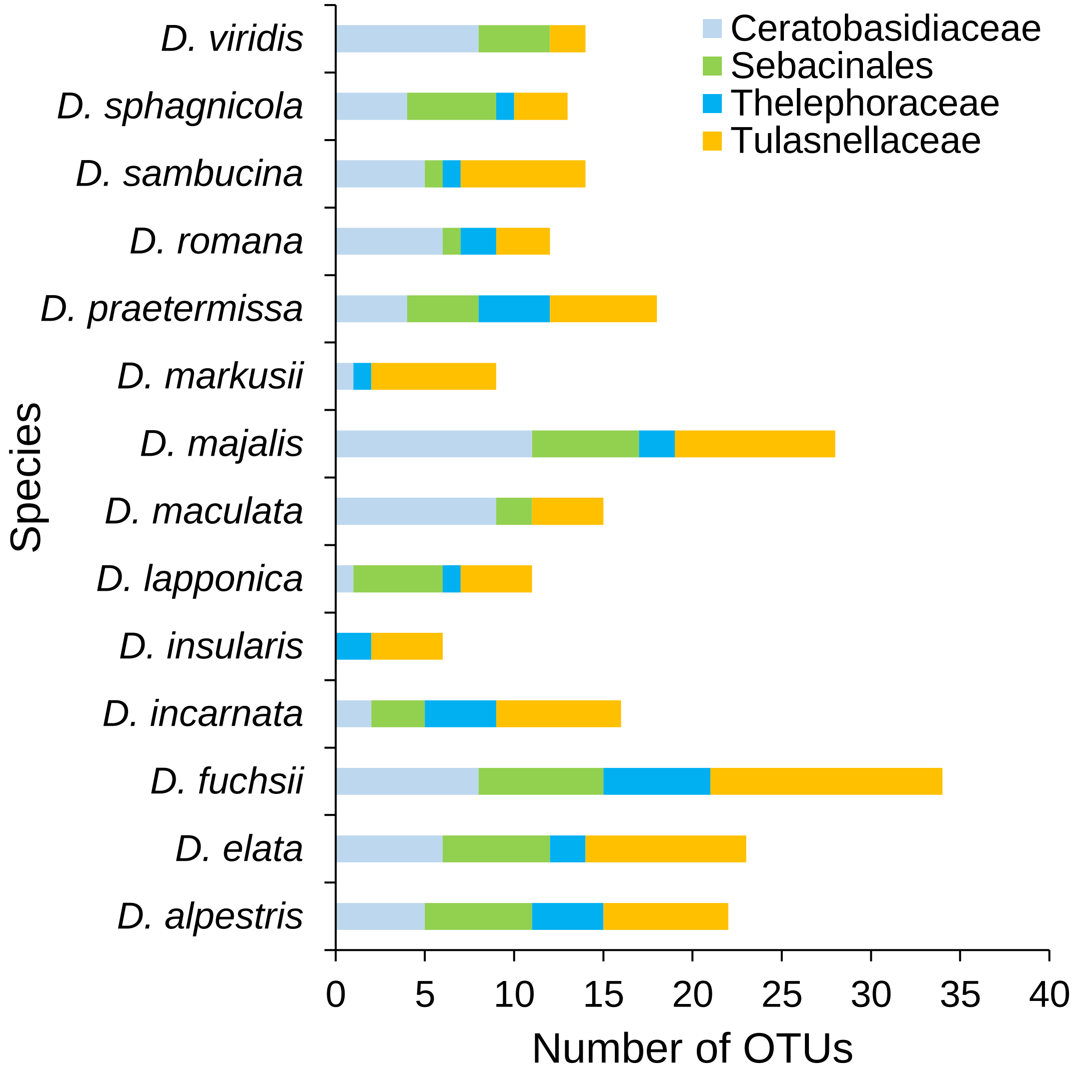
Ceratobasidiaceae
Sebacinales
Thelephoraceae
Tulasnellaceae
D. viridis
D. sphagnicola
D. sambucina
D. romana
D. praetermissa
D. markusii
D. majalis
D. maculata
D. lapponica
D. insularis
D. incarnata
D. fuchsii
D. elata
D. alpestris
Species
0
5
10
15
20
25
30
35
40
Number of OTUs

Fig. S6

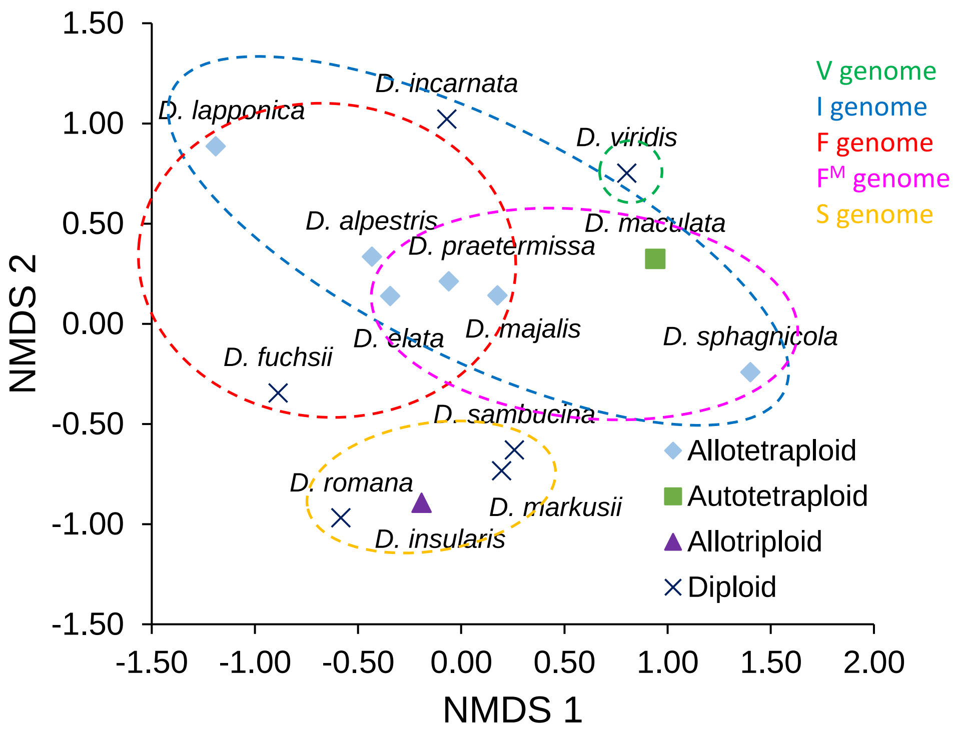

1.50
1.00
0.50
0.00
-0.50
-1.00
-1.50
NMDS 2
-1.50
-1.00
-0.50
0.00
0.50
1.00
1.50
2.00
NMDS 1
D. incarnata
D. lapponica
D. viridis
D. alpestris
D. maculata
D. praetermissa
D. elata
D. majalis
D. sphagnicola
D. fuchsii
D. sambucina
D. romana
D. markusii
D. insularis
V genome
I genome
F genome
F^M genome
S genome
Allotetraploid
Autotetraploid
Allotriploid
Diploid

Fig. S7

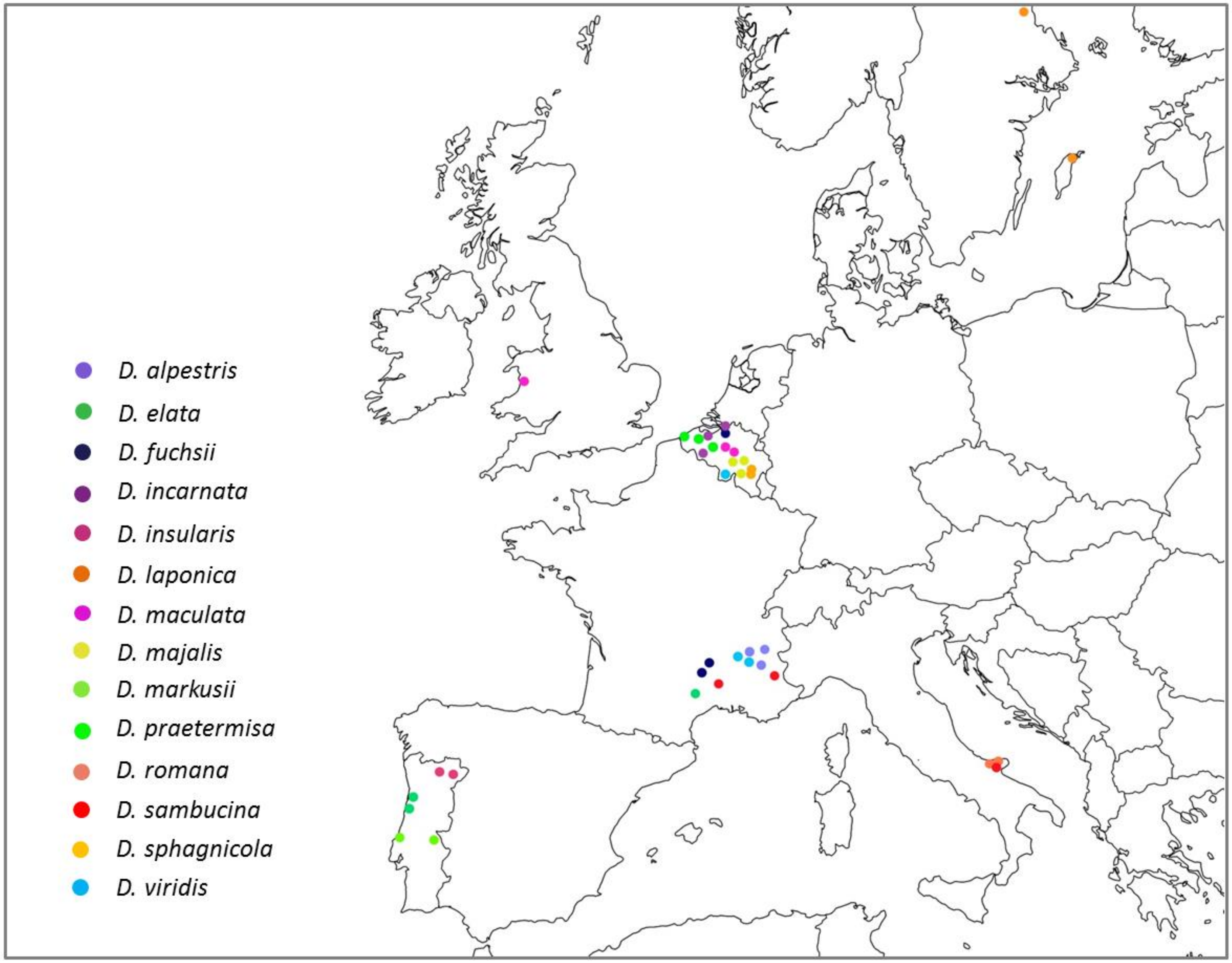
D. alpestris
D. elata
D. fuchsii
D. incarnata
D. insularis
D. laponica
D. maculata
D. majalis
D. markusii
D. praetermisa
D. romana
D. sambucina
D. sphagnicola
D. viridis

Table S1

OTU Id	# sequences	Family	Species	Length	Score	E value	Sequence identity
**OTU 8**	6149	Tulasnellaceae	Uncultured Tulasnella clone	300	532.9	3.9E-148	98.7%
**OTU 4**	5929	Tulasnellaceae	Uncultured mycorrhiza (Tulasnellaceae)	311	442.5	6.8E-121	92.9%
**OTU 10**	4620	Tulasnellaceae	Uncultured mycorrhiza (Tulasnellaceae)	301	505.3	8.6E-140	97.0%
**OTU 60**	4285	Tulasnellaceae	Uncultured mycorrhiza (Tulasnellaceae)	312	475.7	6.7E-131	94.9%
**OTU 1**	4245	Sebacinaceae	Uncultured Sebacinales clone	301	497.9	1.4E-137	95.7%
**OTU 5**	3623	Ceratobasidiaceae	Uncultured Ceratobasidiaceae type OTU	302	521.9	8.5E-145	98.0%
**OTU 18**	3223	Tulasnellaceae	Uncultured Tulasnella isolate GA6b	300	549.6	3.9E-153	99.7%
**OTU 3**	3193	Ceratobasidiaceae	Ceratobasidium sp. UAMH 5443 18S ribosomal RNA gene	300	555.1	8.4E-155	100.0%
**OTU 11**	3084	Ceratobasidiaceae	Uncultured Ceratobasidium clone	301	549.6	3.9E-153	99.7%
**OTU 2**	3025	Ceratobasidiaceae	Ceratobasidium sp. AG-C genes for ITS1	273	468.3	1.1E-128	97.8%
**OTU 9**	2728	Tulasnellaceae	Uncultured Tulasnella clone	300	549.6	3.9E-153	99.7%
**OTU 7**	2063	Tulasnellaceae	Uncultured Tulasnellaceae clone	294	401.8	1.2E-108	91.5%
**OTU 16**	1615	Tulasnellaceae	Uncultured Tulasnella clone	288	405.5	9.0E-110	92.4%
**OTU 17**	1615	Tulasnellaceae	Uncultured mycorrhiza (Tulasnellaceae)	313	425.8	6.9E-116	92.0%
**OTU 21**	1451	Ceratobasidiaceae	Ceratobasidium sp. 2 MV-2011	301	455.4	8.7E-125	94.0%
**OTU 15**	1310	Sebacinaceae	Uncultured Sebacinales clone	300	547.7	1.4E-152	99.7%
**OTU 24**	1027	Tulasnellaceae	Uncultured Tulasnellaceae clone	293	473.9	2.4E-130	95.9%
**OTU19**	885	Sebacinaceae	Uncultured Sebacina mycobiont	301	499.7	4.0E-138	96.7%
**OTU 27**	866	Ceratobasidiaceae	Uncultured Ceratobasidium clone	290	448.0	1.5E-122	94.8%
**OTU 52**	768	Tulasnellaceae	Uncultured Tulasnella clone	277	483.1	4.0E-133	97.5%
**OTU 23**	765	Tulasnellaceae	Uncultured Tulasnellaceae isolate	271	309.5	7.2E-81	88.2%
**OTU 25**	730	Tulasnellaceae	Uncultured mycorrhiza (Tulasnellaceae)	302	442.5	6.8E-121	93.4%
**OTU 22**	700	Thelephoraceae	Uncultured Thelephoraceae clone	286	514.5	1.4E-142	99.0%
**OTU 26**	617	Ceratobasidiaceae	Uncultured Ceratobasidiaceae clone OpaBN01-aab93e01	296	451.7	1.1E-123	94.6%
**OTU 32**	530	Sebacinaceae	Uncultured Sebacina mycobiont	270	475.7	6.7E-131	98.1%
**OTU 31**	360	Tulasnellaceae	Uncultured Tulasnellaceae clone	291	516.3	3.9E-143	98.6%
**OTU 43**	348	Inocybaceae	Uncultured fungus isolate	272	462.8	5.2E-127	97.4%

**OTU 308**	337	Tulasnellaceae	Uncultured Tulasnella clone	281	473.9	2.4E-130	96.8%
**OTU 49**	336	Tulasnellaceae	Uncultured Tulasnellaceae isolate	271	438.8	8.8E-120	95.9%
**OTU 57**	312	Ceratobasidiaceae	Uncultured Rhizoctonia isolate	291	527.4	1.8E-146	99.3%
**OTU 35**	306	Clavulinaceae	Uncultured Agaricales isolate	283	436.9	3.2E-119	95.1%
**OTU 76**	305	Tulasnellaceae	Uncultured mycorrhiza (Tulasnellaceae)	296	532.9	3.9E-148	98.7%
**OTU 48**	299	Ceratobasidiaceae	Ceratobasidium sp.	304	518.2	1.1E-143	97.7%
**OTU 36**	291	Ceratobasidiaceae	Uncultured Ceratobasidiaceae isolate	301	464.6	1.5E-127	94.7%
**OTU 61**	257	Tulasnellaceae	Uncultured Tulasnellaceae clone	311	322.4	9.3E-85	86.2%
**OTU 55**	252	Tulasnellaceae	Uncultured Tulasnellaceae clone	289	492.3	6.7E-136	96.3%
**OTU 140**	246	Tulasnellaceae	Uncultured mycorrhiza (Tulasnellaceae)	311	470.2	3.1E-129	94.5%
**OTU 50**	221	Ceratobasidiaceae	Uncultured ectomycorrhiza (Ceratobasidiaceae)	273	490.5	2.4E-135	98.6%
**OTU 581**	204	Tulasnellaceae	Uncultured mycorrhiza (Tulasnellaceae)	303	540.3	2.4E-150	99.0%
**OTU 46**	202	Ceratobasidiaceae	Ceratobasidium sp.	291	532.9	3.9E-148	99.7%
**OTU 289**	194	Ceratobasidiaceae	Uncultured Ceratobasidiaceae isolate	303	466.5	4.1E-128	94.7%
**OTU 62**	189	Thelephoraceae	Uncultured ectomycorrhiza (Thelephoraceae)	300	532.9	3.9E-148	98.7%
**OTU 72**	188	Tulasnellaceae	Uncultured Tulasnellaceae isolate	278	514.5	1.4E-142	100.0%
**OTU 56**	176	Tulasnellaceae	Uncultured Tulasnellaceae clone	295	440.6	2.5E-120	93.9%
**OTU 79**	176	Armillaria	Uncultured fungus clone	304	411.1	1.9E-111	91.8%
**OTU 67**	163	Sebacinaceae	Uncultured Sebacina clone	300	544.0	1.8E-151	99.3%
**OTU 53**	150	Ceratobasidiaceae	Ceratobasidium sp.	262	348.3	1.5E-92	91.6%
**OTU 82**	130	Ceratobasidiaceae	Ceratobasidium sp.	291	538.5	8.5E-150	100.0%
**OTU 96**	130	Sebacinaceae	Uncultured Sebacina isolate	301	470.2	3.1E-129	95.0%
**OTU 63**	129	Ceratobasidiaceae	Uncultured Basidiomycota clone	300	549.5	3.9E-153	99.7%
**OTU 174**	116	Ceratobasidiaceae	Ceratobasidium sp. AG-Ba isolate	309	350.1	4.3E-93	88.7%
**OTU 81**	107	Ceratobasidiaceae	Uncultured Ceratobasidiaceae clone	302	473.9	2.4E-130	95.0%
**OTU 80**	106	Sebacinaceae	Uncultured Sebacina	297	459.1	6.8E-126	94.9%
**OTU 77**	90	Sebacinaceae	Sebacina vermifera partial 18S rRNA	301	499.7	4.0E-138	96.7%
**OTU 75**	89	Ceratobasidiaceae	Uncultured Rhizoctonia	300	555.1	8.4E-155	100.0%
**OTU 91**	79	Ceratobasidiaceae	Ceratobasidium sp.	301	460.9	1.9E-126	94.4%
**OTU 106**	76	Tulasnellaceae	Uncultured Tulasnellaceae clone	291	538.5	8.5E-150	100.0%

**OTU 92**	68	Sebacinaceae	Uncultured Sebacinales clone	279	505.3	8.6E-140	99.3%
**OTU 102**	66	Ceratobasidiaceae	Uncultured Ceratobasidiaceae isolate	304	472.0	8.7E-130	95.1%
**OTU 123**	65	Clavulinaceae	Uncultured Agaricomycetes clone	274	435.1	1.1E-118	95.6%
**OTU 112**	62	Cortinariaceae	Uncultured Cortinarius clone	300	549.6	3.9E-153	99.7%
**OTU 133**	61	Sebacinaceae	Uncultured Sebacina isolate	300	555.1	8.4E-155	100.0%
**OTU 125**	60	Sebacinaceae	Sebacina vermifera strain	308	381.5	1.5E-102	89.6%
**OTU 144**	56	Tulasnellaceae	Uncultured Tulasnella voucher	300	549.6	3.9E-153	99.7%
**OTU 176**	48	Tulasnellaceae	Uncultured Tulasnellaceae clone	292	529.3	5.1E-147	99.3%
**OTU 104**	42	Inocybaceae	Inocybe petiginosa	300	555.1	8.7E-130	100.0%
**OTU 138**	38	Agaricaceae	Coprinopsis argentea strain	260	468.3	1.1E-128	99.2%
**OTU 161**	37	Clavulinaceae	Uncultured Agaricales isolate	279	516.3	3.9E-143	100.0%
**OTU 156**	35	Marasmius	Uncultured ascomycete clone	300	544.0	1.8E-151	99.3%
**OTU 162**	35	Sebacinaceae	Sebacina vermifera isolate	303	512.6	5.1E-142	97.4%
**OTU 165**	34	Thelephoraceae	Uncultured Thelephoraceae	272	507.1	2.4E-140	97.9%
**OTU 135**	31	Ceratobasidiaceae	Uncultured Ceratobasidium clone	300	549.6	3.9E-153	99.7%
**OTU 169**	28	Sebacinaceae	Uncultured Sebacinales clone	276	505.3	8.6E-140	99.6%
**OTU 136**	25	Ceratobasidiaceae	Ceratobasidium sp.	280	466.5	4.1E-128	96.8%
**OTU 171**	24	Inocybaceae	Uncultured ectomycorrhizal fungus clone	300	542.2	6.6E-151	99.3%
**OTU 146**	23	Thelephoraceae	Uncultured Tomentella genomic DNA	300	555.1	8.4E-155	100.0%
**OTU 240**	22	Ceratobasidiaceae	Thanatephorus cucumeris	300	453.5	3.2E-124	94.3%
**OTU 158**	21	Ceratobasidiaceae	Uncultured Ceratobasidiaceae clone	301	521.9	8.5E-145	98.0%
**OTU 222**	19	Thelephoraceae	Uncultured ectomycorrhizal fungus clone	300	533.0	3.9E-148	98.7%
**OTU 224**	19	Thelephoraceae	Thelephoraceae sp.	301	527.4	1.8E-146	98.3%
**OTU 270**	19	Tulasnellaceae	Uncultured Tulasnellaceae clone	288	516.3	4.0E-143	99.0%
**OTU 191**	18	Sebacinaceae	Uncultured Sebacina mycobiont	300	532.9	3.9E-148	98.0%
**OTU 195**	18	Thelephoraceae	Uncultured ectomycorrhiza (Thelephoraceae)	300	555.1	8.4E-155	100.0%
**OTU 272**	17	Thelephoraceae	Uncultured Tomentella genomic DNA	300	544.0	1.8E-151	99.3%
**OTU 345**	17	Ceratobasidiaceae	Ceratobasidium sp. AG-A isolate	291	533.0	3.9E-148	99.7%
**OTU 159**	16	Psathyrellaceae	Psathyrella sp.	302	424.0	2.5E-115	92.4%
**OTU 249**	15	Ceratobasidiaceae	Ceratobasidium sp.	301	499.7	4.0E-138	96.7%

**OTU 302**	15	Thelephoraceae	Uncultured Thelephoraceae clone	301	549.6	3.9E-153	99.7%
**OTU 181**	14	Tricholomataceae	Uncultured Mycena clone	302	499.7	4.0E-138	96.7%
**OTU 275**	14	Tulasnellaceae	Uncultured Tulasnella mycobiont	300	532.9	3.9E-148	98.7%
**OTU 178**	13	Thelephoraceae	Uncultured ectomycorrhiza (Tomentella)	280	518.2	1.1E-143	100.0%
**OTU 223**	13	Inocybaceae	Uncultured Inocybe genomic DNA	300	555.1	8.4E-155	100.0%
**OTU 168**	12	Thelephoraceae	Uncultured ectomycorrhiza (Thelephoraceae)	300	532.9	3.9E-148	98.7%
**OTU 209**	12	Sebacinaceae	Uncultured Sebacinales clone	273	457.2	2.4E-125	97.1%
**OTU 210**	12	Inocybaceae	Inocybe malenconii	300	555.1	8.4E-155	100.0%
**OTU 241**	12	Sebacinaceae	Uncultured Sebacina isolate	300	555.1	8.4E-155	100.0%
**OTU 314**	12	Ceratobasidiaceae	Uncultured Ceratobasidiaceae isolate	300	525.6	6.6E-140	98.3%
**OTU 399**	12	Thelephoraceae	Tomentella sp. ECM 21	300	510.8	1.8E-148	97.3%
**OTU 199**	11	Thelephoraceae	Uncultured ectomycorrhiza (Thelephoraceae)	301	509.0	6.6E-141	97.3%
**OTU 247**	11	Sebacinaceae	Uncultured Sebacina clone	301	527.4	1.8E-146	98.3%
**OTU 269**	11	Tricholomataceae	Mycena maurella voucher	300	544.0	1.8E-151	99.3%
**OTU 297**	10	Clavulinaceae	Uncultured Agaricomycetes clone	275	466.5	4.1E-128	97.5%
**OTU 250**	9	Sebacinaceae	Uncultured Sebacina isolate	279	494.2	1.9E-136	98.6%
**OTU 439**	9	Atractiella	Uncultured endophytic fungus clone	188	348.3	1.5E-92	100.0%
**OTU 232**	8	Sebacinaceae	Uncultured Sebacina mycobiont	305	464.6	1.5E-127	94.4%
**OTU 366**	8	Agaricaceae	Uncultured soil basidiomycete isolate	301	549.6	3.9E-153	99.7%
**OTU 395**	8	Inocybaceae	Uncultured Inocybe genomic DNA	300	531.1	1.4E-147	98.7%
**OTU 519**	8	Psathyrellaceae	Psathyrella typhae	300	529.3	5.1E-147	98.7%
**OTU 287**	7	Sebacinaceae	Uncultured Sebacinaceae clone	273	418.5	1.2E-113	94.5%
**OTU 414**	7	Clavulinaceae	Uncultured fungus clone	303	457.2	2.4E-125	94.4%
**OTU 316**	6	Tulasnellaceae	Uncultured Tulasnellaceae clone	160	296.6	5.6E-77	100.0%
**OTU 357**	6	Ceratobasidiaceae	Uncultured Ceratobasidiaceae isolate	302	436.9	3.2E-119	93.0%
**OTU 370**	6	Cortinariaceae	Uncultured ectomycorrhiza (Cortinarius) clone	302	510.8	1.8E-141	97.4%
**OTU 273**	5	Thelephoraceae	Uncultured ectomycorrhiza (Tomentella) clone	291	514.5	1.4E-142	98.6%
**OTU 274**	5	Tricholomataceae	Uncultured Cortinarius genomic DNA	300	529.3	5.1E-147	98.7%
**OTU 280**	5	Clavulinaceae	Uncultured ectomycorrhiza (Clavulinaceae) clone	302	529.3	5.1E-147	98.3%
**OTU 286**	5	Thelephoraceae	Uncultured Tomentella genomic DNA	300	555.1	8.4E-155	100.0%

**OTU 336**	5	Sebacinaceae	Uncultured Sebacinaceae clone	250	226.4	7.5E-56	80.4%
**OTU 339**	5	Sebacinaceae	Uncultured Sebacina isolate	300	521.9	8.5E-145	98.0%
**OTU 391**	5	Ceratobasidiaceae	Uncultured Ceratobasidiaceae isolate	294	483.1	4.0E-133	96.6%
**OTU 419**	5	Sebacinaceae	Uncultured Sebacina isolate	300	544.0	1.8E-151	99.3%
**OTU 420**	5	Sebacinaceae	Sebacina vermifera strain	312	374.1	2.5E-100	89.1%
**OTU 433**	5	Sebacinaceae	Uncultured Sebacinales clone	298	470.2	3.1E-129	95.3%
**OTU 495**	5	Tulasnellaceae	Uncultured Tulasnellaceae clone OmiA	291	532.9	3.9E-148	99.7%
**OTU 325**	4	Thelephoraceae	Uncultured ectomycorrhiza (Tomentella) isolate	300	549.6	3.9E-153	99.7%
**OTU 331**	4	Ceratobasidiaceae	Uncultured Ceratobasidiaceae type OTU	301	516.3	4.0E-143	97.7%
**OTU 338**	4	Thelephoraceae	Thelephoraceae sp.	300	533.0	3.9E-148	98.7%
**OTU 385**	4	Sebacinaceae	Uncultured Sebacinales	300	481.2	1.4E-132	95.7%
**OTU 580**	4	Inocybaceae	Inocybe praetervisa isolate	301	549.6	3.9E-153	99.7%
**OTU 371**	3	Sebacinaceae	Uncultured Sebacina isolate	300	540.3	2.4E-150	99.0%
**OTU 394**	3	Sebacinaceae	Uncultured Sebacina mycobiont	300	555.1	8.4E-155	100.0%
**OTU 406**	3	Sebacinaceae	Uncultured Sebacina isolate	300	555.1	8.4E-155	100.0%
**OTU 428**	3	Thelephoraceae	Uncultured ectomycorrhiza (Thelephoraceae)	301	427.7	1.9E-116	92.7%
**OTU 434**	3	Sebacinaceae	Uncultured Sebacina isolate	307	357.5	2.5E-95	88.3%
**OTU 440**	3	Sebacinaceae	Uncultured Sebacina isolate	300	549.6	3.9E-153	99.7%
**OTU 442**	3	Tricholomataceae	Uncultured basidiomycete clone	293	414.7	1.5E-112	92.5%
**OTU 459**	3	Tricholomataceae	Uncultured cf. Mycena sp. clone	300	555.1	8.4E-155	100.0%
**OTU 506**	3	Ceratobasidiaceae	Ceratobasidium sp. UAMH	301	492.3	6.7E-136	96.3%
**OTU 522**	3	Ceratobasidiaceae	Uncultured Ceratobasidiaceae isolate	302	425.8	6.9E-116	92.4%
**OTU 536**	3	Tricholomataceae	Mycena vitilis	300	549.6	3.9E-153	99.7%
**OTU 549**	3	Thelephoraceae	Uncultured Tomentella clone	300	499.7	8.4E-138	98.7%
**OTU 588**	3	Sebacinaceae	Uncultured Sebacina isolate	300	549.6	3.9E-153	99.7%

Table S2

Species	Country	Sampling site	No. Plants sampled
D. alpestris	France	Col Bayard	3
	France	Col du Lautaret 1	3
	France	Col du Lautaret 2	3
D. elata	France	Avène	3
	Portugal	Senhora de Vagos	3
	Portugal	Santo André	3
D. fuchsii	France	Meyrueis 1	3
	France	Meyrueis 2	3
	Belgium	Hobokense Polder	3
D. incarnata	Belgium	Westhoek	3
	Belgium	Ekers Moeras	3
	Belgium	Vaarttaluds (Moen)	3
D. insularis	Portugal	Mogadouro	3
	Portugal	Macedo de Cavaleiros	3
D. lapponica	Sweden	Hälsningland, Loos parish, locality Långtjärnen	3
	Sweden	Gotland, Rute parish, Kauparve	3
D. maculata	Belgium	Papendel 1	3
	Belgium	Vorsdonkbos	3
	UK (Wales)	Edem	3
D. majalis	Belgium	Aardgat (Tienen)	3
	Belgium	Malendriesbeekvallei (Kerkom)	3
	Belgium	Revogne	3
D. markusii	Portugal	Carvalhal	3
	Portugal	Castelo de Vide	3
D. praetermissa	Belgium	Leiemeeersen	3
	Belgium	Gavers Harelbeke	3
	Belgium	Warandeduinen (Middelkerke)	3

D. romana	Italy	Gargano3	3
	Italy	Cagnano Varano	3
D. sambucina	France	L'Esperou	3
	France	Merlette-Orcieres	3
	Italy	Monte Calvo	3
D. sphagnicola	Belgium	St. Hubert 1	3
	Belgium	St. Hubert 2	3
	Belgium	Warinsart	3
D. viridis	France	Col de Menée	3
	France	Romeyer	3
	Belgium	Bonnerieu	3
		Total	114
